# Hepato-renal protection by ferulic acid in a type 2 diabetic rat model: *in vivo* and *in silico* insights into carbohydrate metabolism, REDOX balance, and inflammation modulation

**DOI:** 10.5114/bta/207911

**Published:** 2025-09-07

**Authors:** Jude Akinyelu, Akinwunmi Oluwaseun Adeoye, Chika Ifeanyi Chukwuma, Toluwase Hezekiah Fatoki, Olufunbi Kehinde Adewumi, Ikenna Maximillian Odoh, Gift Racheal Ekun, Abidemi Sherifdeen Adeleke

**Affiliations:** 1 Department of Biochemistry, Federal University Oye-Ekiti, Ekiti State, Nigeria; 2 Biomembrane, Phytomedicine, and Drug Development Unit, Department of Biochemistry, Federal University Oye-Ekiti, Ekiti State, Nigeria; 3 Centre for Quality of Health and Living, Faculty of Health and Environmental Sciences, Central University of Technology, Bloemfontein, South Africa; 4 University Medical Centre, Federal University Oye-Ekiti, Ekiti State, Nigeria

**Keywords:** diabetes, liver, kidney, antioxidant, inflammation, therapeutic

## Abstract

**Background:**

Type 2 diabetes (T2D) is a global health concern characterized by pancreatic β-cell dysfunction, which disrupts multiple biochemical pathways. Consequently, treatments that target various pathways are essential. This study evaluates the hepato-renal protective effects of ferulic acid (FA) in T2D, focusing on carbohydrate metabolism, oxidative stress, and inflammation using *in vivo* and *in silico* approaches.

**Materials and methods:**

T2D was induced in male Wistar rats using fructose and streptozotocin. After 28 days of FA treatment, biochemical analyses were performed to measure glucose, glycosylated hemoglobin, insulin, liver enzymes (ALT, AST, ALP), renal markers (creatinine, uric acid, BUN), and antioxidant status (SOD, CAT, GSH, MDA) in the liver and kidney. Pro-inflammatory markers (NF-κB-p65, IL-1β, IL-6) were evaluated in the liver and kidney. Molecular docking studies were also conducted to assess FA’s interaction with key T2D-related proteins.

**Results:**

FA treatment improved pancreatic β-cell function, increased insulin levels, and reduced serum glucose and glycosylated hemoglobin. Liver function, renal markers, and hepatic glycogen content improved significantly, and diabetes-induced weight loss was reversed. FA inhibited pancreatic α-amylase, intestinal α-glucosidase, fructose-1,6-bisphosphatase, and glucose-6-phosphatase, while enhancing hexokinase activity. Notably, FA improved antioxidant status and reduced inflammatory mediators in diabetic rats. Molecular docking revealed that FA exhibits stronger binding affinity and greater inhibitory potential against key diabetes-related proteins compared to metformin.

**Conclusion:**

FA offers hepato-renal protection in T2D by modulating carbohydrate metabolism, oxidative stress, and inflammation, highlighting its potential as a therapeutic agent against T2D.

## Introduction

Diabetes mellitus is a metabolic disorder characterized by elevated blood glucose levels and has become a critical global health concern. The disease arises from either reduced insulin secretion by pancreatic β-cells (type 1 diabetes) or impaired tissue responsiveness to insulin (type 2 diabetes [T2D]) (Kumar et al. 2020). Uncontrolled hyperglycemia in diabetic patients leads to significant disruptions in the metabolism of carbohydrates, proteins, and lipids (González et al. 2023). This metabolic dysregulation, particularly under conditions of persistent hyperglycemia, results in widespread physiological and biochemical consequences.

In addition to metabolic disturbances, sustained hyperglycemia is linked to excessive generation of free radicals, which can overwhelm the body’s antioxidant defenses and lead to oxidative stress (González et al. 2023). Oxidative stress, in turn, is a major activator of nuclear factor kappa-light-chain-enhancer of activated B cells-p65 (NF-κB-p65), a key inflammatory mediator that induces the production of pro-inflammatory proteins such as interleukin-1 beta (IL-1β) and interleukin-6 (IL-6). These cytokines are frequently implicated in diabetes-related inflammatory complications (Plowman et al. 2023).

The liver and kidney are particularly susceptible to oxidative insults, which are exacerbated under diabetic conditions (Dab et al. 2023). Therefore, enhancing the body’s antioxidant defense system is crucial for mitigating oxidative stress and inflammation-induced damage to these organs.

Although conventional chemical and synthetic medications for T2D are available, their high cost and potential toxicity with prolonged use pose significant challenges (Kanwal et al. 2022). This highlights the need for alternative treatments that are cost-effective, safe, and capable of achieving glucose homeostasis, enhancing insulin sensitivity, reducing oxidative stress, and mitigating inflammation. Given the structural diversity and biological richness of plants, many plant-derived compounds have demonstrated remarkable pharmacological potential, spurring interest in identifying novel plant-based or synthetic analogs for treating T2D (Chaachouay and Zidane 2024).

Ferulic acid (FA), a bioactive compound found in oats, pineapples, peanuts, wheat, and other sources, has been reported to possess various pharmacological properties, including antidiabetic (Salau et al. 2023), antioxidant (Liu et al. 2022), anti-inflammatory (Liu et al. 2022), cardioprotective (Pandi et al. 2022), and hypolipidemic (Zeng et al. 2024) effects.

Molecular docking has become a critical tool in drug development, enabling researchers to predict the interactions between small molecules and biological target proteins at the structural level (Muhammed and Aki-Yalcin 2024). This technique helps elucidate the biochemical processes underlying these interactions and provides useful insights into the behavior of bioactive compounds within the active sites of target proteins in a simulated biological environment.

The aim of this study is to explore the modulatory potential of FA on carbohydrate metabolism, oxidative stress, and inflammation in T2D using both *in vivo* and *in silico* approaches. This will provide insights into the underlying mechanisms of ferulic acid’s protective effects and its potential as an antidiabetic agent.

## Materials and Methods

### Materials

All chemicals and reagents used for this study are of analytical grade.

### Experimental design and animal treatment

This study strictly followed the National Institutes of Health guidelines for the care and use of laboratory animals (NIH Publication No. 8523, revised 2011). All protocols were approved by the Research Ethics Committee of the Federal University Oye-Ekiti, Ekiti State, Nigeria.

Thirty (30) male Wistar rats (140–160 g) were obtained from the experimental rat-breeding unit at Ekiti State University, Nigeria. The animals were acclimatized for 14 days in clean wire cages at room temperature (25 ± 2°C) with a 12-h light-dark cycle and provided with rat feed and clean water *ad libitum*.

Following acclimatization, the rats received 10% fructose (w/v) in drinking water *ad libitum* for 2 weeks to induce insulin resistance. Subsequently, streptozotocin (STZ; 40 mg/kg body weight), freshly prepared in 0.1 M citrate buffer (pH 4.5), was administered intraperitoneally to induce partial pancreatic β-cell dysfunction. Blood glucose levels were monitored using an Accu-Chek glucometer (Roche Diagnostics, Germany). Rats with blood glucose levels exceeding 200 mg/dl after 72 h were considered diabetic and were randomly divided into six groups (five rats per group), as follows:

- Group 1 (NC): normal control,
- Group 2 (DC): diabetic control,
- Group 3 (DFA 25 mg/kg): diabetic rats administered 25 mg/kg bw FA,
- Group 4 (DFA 50 mg/kg): diabetic rats administered 50 mg/kg bw FA,
- Group 5 (FA 50 mg/kg): normal rats administered 50 mg/kg bw FA,
- Group 6 (DBMET): diabetic rats administered 200 mg/kg bw metformin.

FA was administered daily via oral gavage for 28 days. Fasting blood glucose levels were measured at intervals of at least 3 days. On the final day of treatment, the fasting blood glucose levels and body weights of overnightfasted rats were recorded. The rats were then humanely sacrificed, and blood samples were collected via cardiac puncture and centrifuged to separate the serum.

The liver, kidney, pancreas, and small intestine were carefully dissected, rinsed with cold phosphate-buffered saline, blotted dry, and weighed. The organ samples were then homogenized and stored in a bio-freezer for subsequent analyses.

### Rationale for dose selection

In this study, the concentration of fructose (10%) and the dose of STZ (40 mg/kg bw) were selected based on an established protocol for the induction of T2D, with slight modifications (Wilson and Islam 2012). The treatment doses of FA (25 and 50 mg/kg bw) were adopted based on a previous study (Chowdhury et al. 2019). Additionally, 200 mg/kg bw of metformin was selected as the standard drug treatment dose, as reported in a prior study (Quaile et al. 2010).

### Biochemical analyses

Rat ELISA kits (Wuhan Fine Biotech Co., China) were used to determine serum insulin concentration and glycated hemoglobin levels. Total protein in the serum was measured using a previously described method (Gornall et al. 1949). Liver function markers – including alanine aminotransferase (ALT), aspartate aminotransferase (AST), and alkaline phosphatase (ALP) activities – were assessed in the serum using the method of Reitman and Frankel (1957). Rat ELISA kits (Wuhan Fine Biotech Co., China) were also employed to measure α-amylase and α-glucosidase activities in the pancreas and small intestine, respectively. Additional carbohydrate-metabolizing enzyme assays were conducted using liver homogenates. These included assessments of hexokinase activity (Brandstrup et al. 1957), glucose-6-phosphatase activity (Baginski et al. 1974), fructose-1,6-bisphosphatase activity (Gancedo and Gancedo 1971), and hepatic glycogen content (Passonneau and Lauderdale 1974).

In liver and kidney homogenates, superoxide dismutase (SOD) activity was determined using the Misra and Fridovich method (1972), catalase activity was measured following the method of Beers and Sizer (1952), reduced glutathione (GSH) concentration was evaluated using the modified Ellman method (Owens and Belcher 1965), and malondialdehyde (MDA) levels were assessed using a modified version of the Yan Li and Chow method (Li and Chow 1994). Furthermore, nuclear factor-kappa B (NF-κB), interleukin-1 beta (IL-1β), and interleukin-6 (IL-6) levels in liver and kidney tissue homogenates were measured using commercially available ELISA kits (Thermo Fisher Scientific, Massachusetts, United States).

### Histological analysis

Liver and kidney tissues fixed in 10% formalin were dehydrated using graded alcohol concentrations ranging from 50% to 70%, followed by clearing with xylene. The tissues were then embedded in paraffin and sectioned at 25 μm and 5 μm, respectively. The sections were placed on slides, drained, and gently heated on a hot plate to melt the paraffin wax and enhance adhesion. The slides were stained with hematoxylin and eosin (H&E), and dibutylphthalate polystyrene xylene (DPX) was used as the mounting medium. Finally, the stained slides were examined under a microscope.

### Molecular docking

The 3D structures of α-amylase (1B2Y), α-glucosidase (3L4Y), fructose-1,6-bisphosphatase (2WBD), glycogen synthase kinase-3β (6H0U), myeloperoxidase (5QJ3), and NF-κB-p65 (1NFI) were obtained from the Protein Data Bank (PDB). The structure of glucose-6-phosphatase was modeled using the SwissModel server (Waterhouse et al. 2018). All target proteins were prepared using the Schrodinger Maestro (v11.1) Protein Preparation Wizard, where structural issues were resolved before optimization and minimization. SiteMap in Schrodinger Maestro was used to identify the top-ranked binding regions, followed by receptor grid generation using Glide.

The 3D structure of FA was downloaded from Pub-Chem (CID: 445858) and prepared using the LigPrep wizard (OPLS3) in Schrodinger Maestro (v11.1). Extra precision (XP) docking was then performed, and the docking scores along with ligand interaction diagrams were generated. The binding energy was estimated using the molecular mechanics with generalized Born and surface area solvation (MMGBSA) method. The inhibition constant (Ki) was calculated using the formula:

Ki=EXP (ΔG/RT),

where Δ*G* is the estimated free binding energy in kcal/mol, *R* is the gas constant (1.987 × 10^–3^ kcal/(mol · K)), and *T* is room temperature (298 K).

### Statistical analysis

Statistical analyses were performed using GraphPad Prism (version 8, USA) by one-way analysis of variance (ANOVA), and results were expressed as mean ± standard error of the mean (SEM). Multiple comparisons between groups were conducted using the Bonferroni post hoc test. Differences were considered statistically significant at *p* < 0.05.

## Results

### Serum glucose and insulin, glycosylated haemoglobin, total protein, and body weight

**Figure 1:**
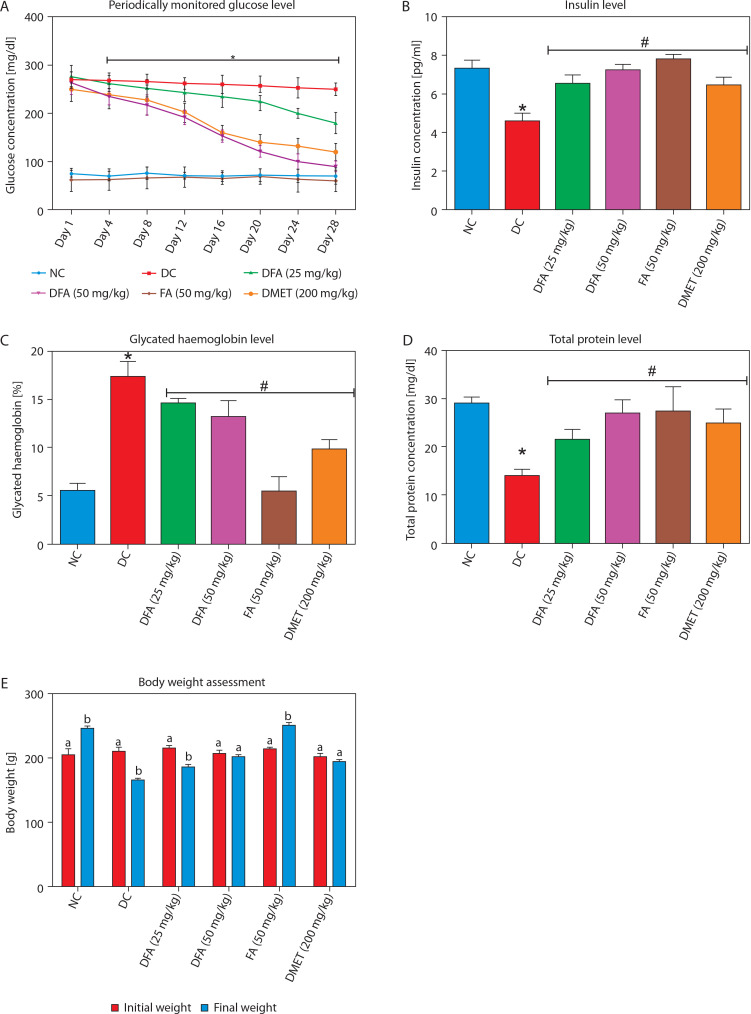
**A)** Fasting blood glucose concentration. **B)** Serum insulin level. **C)** Glycated haemoglobin level. **D)** Total protein. **E)** Body weight. Values are mean ± SEM of five experimental animals (*n* = 5). **A)** Asterisk (*) represents a significant difference (*p* < 0.05) between diabetic control and other groups at each day point measured. **B–D)** Asterisk (*) represents a significant difference (*p* < 0.05) to the NC group, while ^#^ represents a significant difference (*p* < 0.05) to the DC group. **E)** For each group, columns bearing different letters are significantly different (*p* < 0.05) from each other NC – normal control, DC – diabetic control, DFA (25 mg/kg) – diabetic rats treated with 25 mg/kg bw ferulic acid, DFA (50 mg/kg) – diabetic rats treated with 50 mg/kg bw ferulic acid, FA (50 mg/kg) – normal rats administered with 50 mg/kg bw ferulic acid, DMET (200 mg/kg) – diabetic rats treated with 200 mg/kg bw metformin

In diabetic rats treated with FA or metformin, serum glucose (Figure 1A) and glycosylated hemoglobin (Figure 1C) levels were significantly reduced (*p* < 0.05), while insulin (Figure 1B), total protein (Figure 1D), and body weight (Figure 1E) increased significantly (*p* < 0.05) compared to untreated diabetic rats.

### Liver and kidney biomarkers

**Figure 2:**
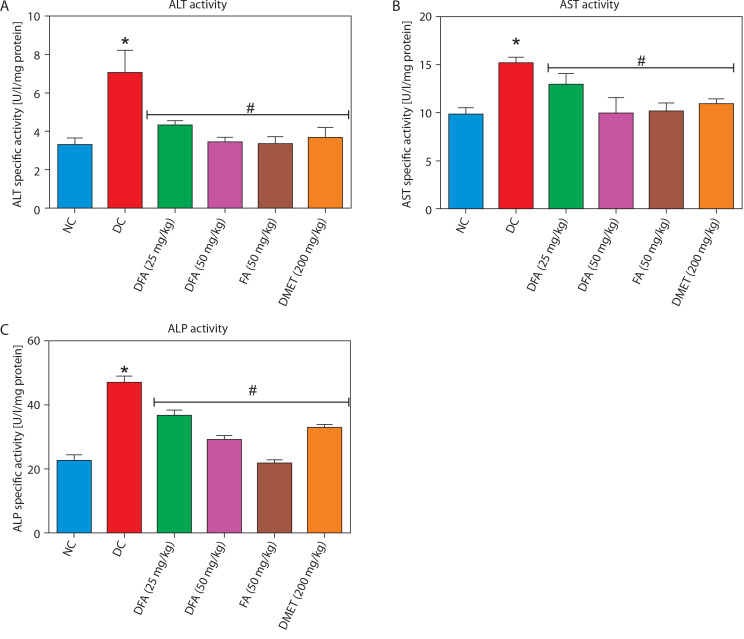
Effect of FA on levels of **(A)** ALT, **(B)** AST, **(C)** ALP. Values are mean ± SEM of five experimental animals (*n* = 5). Asterisk (*) represents a significant difference (*p* < 0.05) to the NC group, while ^#^ represents a significant difference (*p* < 0.05) to the DC group NC – normal control, DC – diabetic control, DFA (25 mg/kg) – diabetic rats treated with 25 mg/kg bw ferulic acid, DFA (50 mg/kg) – diabetic rats treated with 50 mg/kg bw ferulic acid, FA (50 mg/kg) – normal rats administered with 50 mg/kg bw ferulic acid, DMET (200 mg/kg) – diabetic rats treated with 200 mg/kg bw metformin

**Figure 3:**
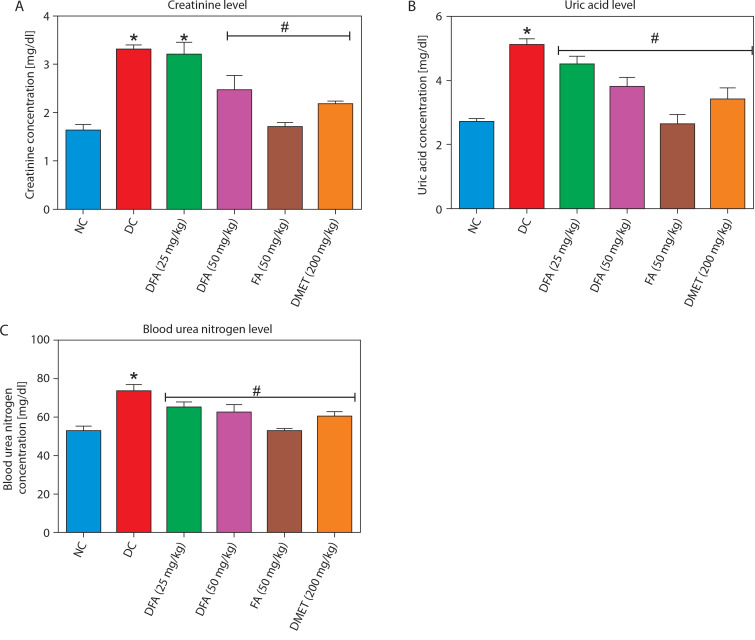
Effect of ferulic acid on (**A**) creatinine, (**B**) uric acid, (**C**) blood urea nitrogen. Values are mean ± SEM of five experimental animals (*n* = 5). Asterisk (*) represents a significant difference (*p* < 0.05) to the NC group, while ^#^ represents a significant difference (*p* < 0.05) to the DC group NC – normal control, DC – diabetic control, DFA (25 mg/kg) – diabetic rats treated with 25 mg/kg bw ferulic acid, DFA (50 mg/kg) – diabetic rats treated with 50 mg/kg bw ferulic acid, FA (50 mg/kg) – normal rats administered with 50 mg/kg bw ferulic acid, DMET (200 mg/kg) – diabetic rats treated with 200 mg/kg bw metformin

As shown in Figure 2A–C, treatment with FA or metformin effectively reduced serum levels of ALT, AST, and ALP in diabetic rats compared to the untreated diabetic group. Similarly, Figure 3A–C indicates that treatment with FA (50 mg/kg bw) or metformin significantly (*p* < 0.05) reduced levels of creatinine, uric acid, and blood urea nitrogen (BUN) compared to untreated diabetic rats.

### Carbohydrate metabolic markers

**Figure 4:**
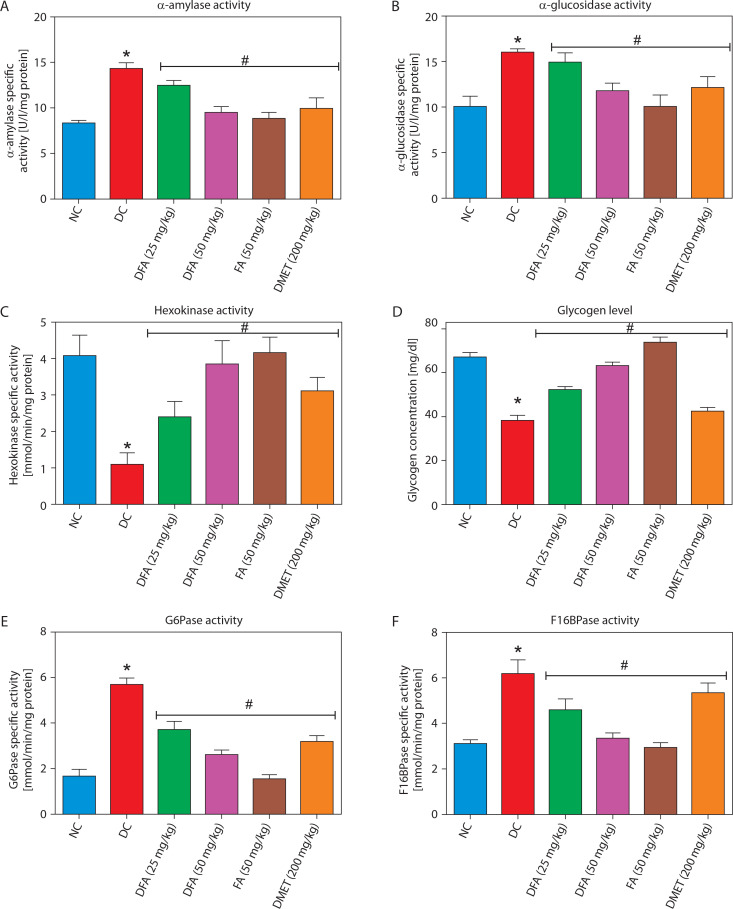
Effect of ferulic acid on specific activity of **(A)** α-amylase, **(B)** α-glucosidase, **(C)** hexokinase, **(D)** hepatic glycogen, **(E)** G6Bpase, and **(F)** F16Bpase. Values are mean ± SEM of five experimental animals (*n* = 5). Asterisk (*) represents a significant difference (*p* < 0.05) to the NC group, while ^#^ represents a significant difference (*p* < 0.05) to the DC group NC – normal control, DC – diabetic control, DFA (25 mg/kg) – diabetic rats treated with 25 mg/kg bw ferulic acid, DFA (50 mg/kg) – diabetic rats treated with 50 mg/kg bw ferulic acid, FA (50 mg/kg) – normal rats administered with 50 mg/kg bw ferulic acid, DMET (200 mg/kg) – diabetic rats treated with 200 mg/kg bw metformin

FA or metformin treatment decreased the activities of pancreatic α-amylase (Figure 4A) and intestinal α-glucosidase (Figure 4B), while enhancing hepatic hexokinase activity (Figure 4C) and glycogen levels (Figure 4D), compared to the untreated diabetic group. In addition, the activities of gluconeogenic enzymes — glucose-6-phosphatase (G6Pase) (Figure 4E) and fructose-1,6-bisphosphatase (F16Bpase) (Figure 4F) – were significantly suppressed in treated diabetic rats.

### Oxidative stress markers in the liver and kidney

**Figure 5:**
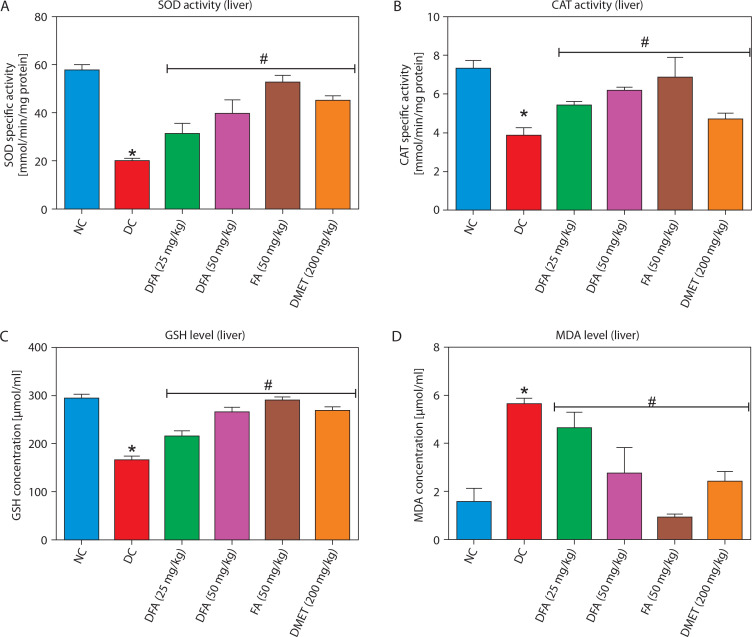
Effect of ferulic acid on specific activities of **(A)** SOD, **(B)** CAT, **(C)** GSH concentration, and **(D)** MDA level in liver. Values are mean ± SEM of five experimental animals (*n* = 5). Asterisk (*) represents a significant difference (*p* < 0.05) to the NC group, while ^#^ represents a significant difference (*p* < 0.05) to the DC group NC – normal control, DC – diabetic control, DFA (25 mg/kg) – diabetic rats treated with 25 mg/kg bw ferulic acid, DFA (50 mg/kg) – diabetic rats treated with 50 mg/kg bw ferulic acid, FA (50 mg/kg) – normal rats administered with 50 mg/kg bw ferulic acid, DMET (200 mg/kg) – diabetic rats treated with 200 mg/kg bw metformin

**Figure 6:**
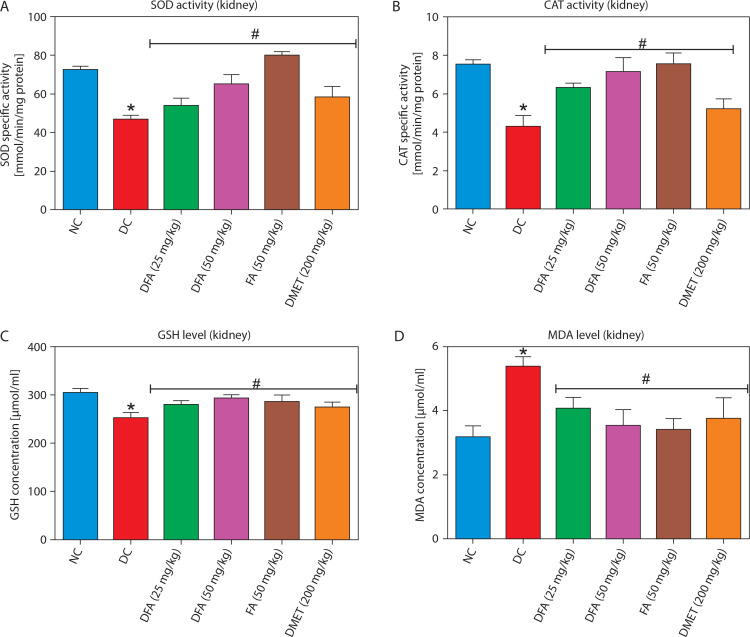
Effect of ferulic acid on specific activities of **(A)** SOD, **(B)** CAT, **(C)** GSH concentration, and **(D)** MDA level in the kidney. Values are mean ± SEM of five experimental animals (*n* = 5). Asterisk (*) represents a significant difference (*p* < 0.05) to the NC group, while ^#^ represents a significant difference (*p* < 0.05) to the DC group NC – normal control, DC – diabetic control, DFA (25 mg/kg) – diabetic rats treated with 25 mg/kg bw ferulic acid, DFA (50 mg/kg) – diabetic rats treated with 50 mg/kg bw ferulic acid, FA (50 mg/kg) – normal rats administered with 50 mg/kg bw ferulic acid, DMET (200 mg/kg) – diabetic rats treated with 200 mg/kg bw metformin

In both the liver (Figure 5A–D) and kidney (Figure 6A–D), SOD and CAT activities, along with GSH levels, were significantly (*p* < 0.05) increased in diabetic rats treated with FA or metformin. Additionally, MDA formation was significantly (*p* < 0.05) reduced compared to the untreated diabetic group.

### Pro-inflammatory factors in the liver and kidney

**Figure 7:**
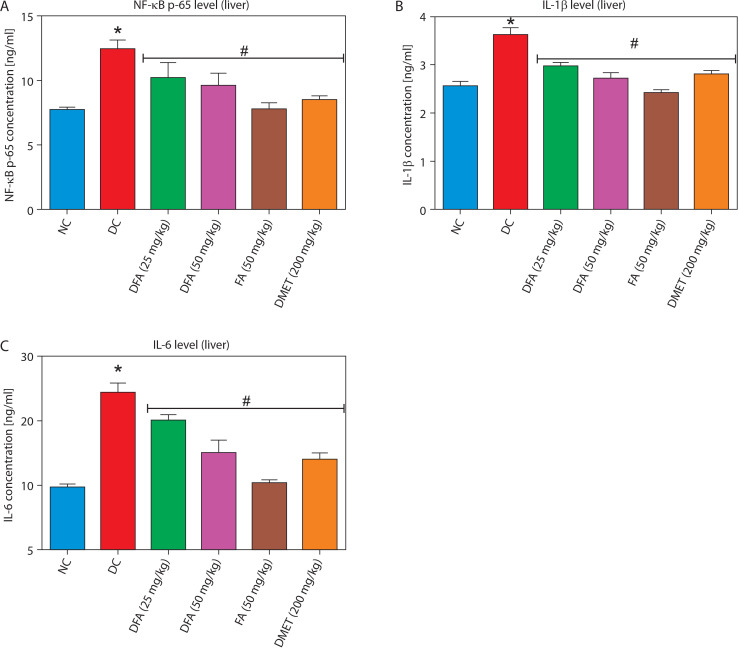
Effect of ferulic acid on **(A)** NF-κB-p65, **(B)** IL-1β, and **(C)** IL-6 protein levels in liver tissue. Values are mean ± SEM of five experimental animals (*n* = 5). Asterisk (*) represents a significant difference (*p* < 0.05) to the NC group, while ^#^ represents a significant difference (*p* < 0.05) to the DC group NC – normal control, DC – diabetic control, DFA (25 mg/kg) – diabetic rats treated with 25 mg/kg bw ferulic acid, DFA (50 mg/kg) – diabetic rats treated with 50 mg/kg bw ferulic acid, FA (50 mg/kg) – normal rats administered with 50 mg/kg bw ferulic acid, DMET (200 mg/kg) – diabetic rats treated with 200 mg/kg bw metformin

**Figure 8:**
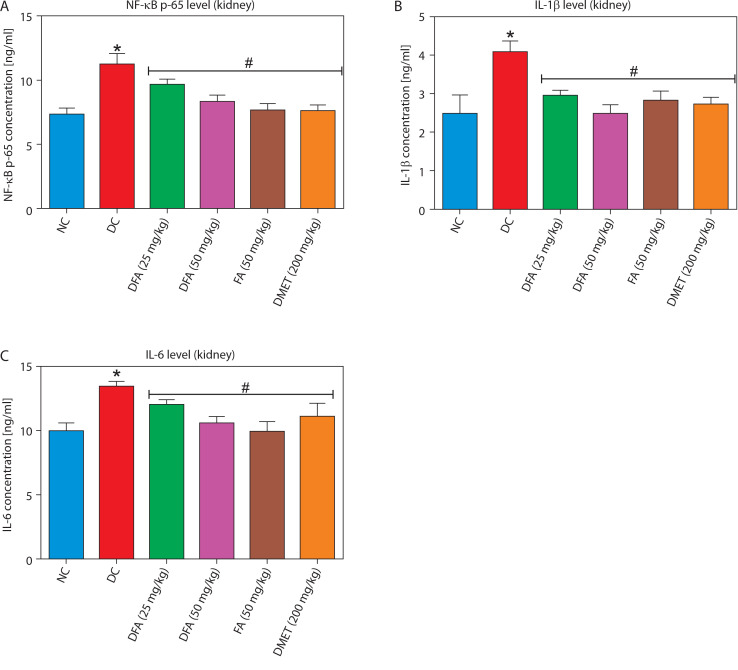
Effect of ferulic acid on **(A)** NF-κB-p65, **(B)** IL-1β, and **(C)** IL-6 protein levels in kidney tissue. Values are mean ± SEM of five experimental animals (*n* = 5). Asterisk (*) represents a significant difference (*p* < 0.05) to the NC group, while ^#^ represents a significant difference (*p* < 0.05) to the DC group NC – normal control, DC – diabetic control, DFA (25 mg/kg) – diabetic rats treated with 25 mg/kg bw ferulic acid, DFA (50 mg/kg) – diabetic rats treated with 50 mg/kg bw ferulic acid, FA (50 mg/kg) – normal rats administered with 50 mg/kg bw ferulic acid, DMET (200 mg/kg) – diabetic rats treated with 200 mg/kg bw metformin

Treatment with FA or metformin significantly (*p* < 0.05) reduced the levels of pro-inflammatory mediators – NF-κB-p65, IL-1β, and IL-6 – in the liver (Figure 7A–D) and kidney (Figure 8A–D) compared to untreated diabetic rats.

### Histopathological analysis of the liver and kidney

As illustrated in the representative photomicrograph images (Supplementary Figure 1A–F), liver tissue from the normal control group showed no blood congestion and moderate vacuolation with no visible lesions. In contrast, liver tissue from the untreated diabetic group displayed congestion, dilation of the central vein, and nuclear fragmentation. Notably, groups treated with FA or metformin showed only mild congestion, limited nuclear fragmentation, and moderate loss of hepatocytes.

Similarly, the representative photomicrograph images (Supplementary Figure 2A–F) show that kidney tissue from the normal control group exhibited normal architecture of the glomerulus and convoluted tubules with no visible lesions. In contrast, the untreated diabetic group displayed degeneration of the glomerulus, enlargement of the urinary space, and degeneration of convoluted tubule cells. These pathological changes were significantly moderated upon treatment with FA.

### Molecular docking analyses

#### Carbohydrate metabolising enzymes

**Figure 9:**
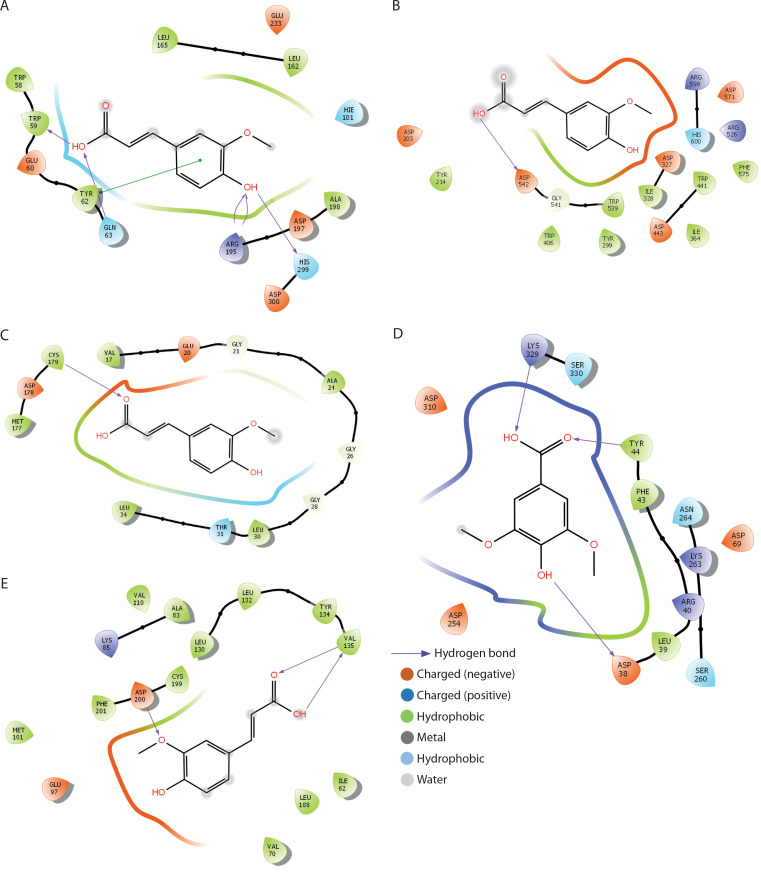
2D binding interaction between ferulic acid and carbohydrate metabolising enzymes. **A**) α-amylase. **B**) α-glucosidase. **C**) fructose-1,6-bisphosphatase. **D**) glucose-6-phosphatase. E) glycogen synthase kinase-3β

The binding interactions of FA with α-amylase, α-glucosidase, fructose-1,6-bisphosphatase, glucose-6-phosphatase, and glycogen synthase kinase-3β are summarized in Table 1 and Figure 9A–D. FA exhibited strong binding affinities, with docking scores of –6.5, –4.1, –5.7, –5.1, and –7.6 kcal/mol for α-amylase, α-glucosidase, fructose-1,6-bisphosphatase, glucose-6-phosphatase, and glycogen synthase kinase-3β, respectively — each significantly outperforming metformin. The estimated free binding energies for FA with these enzymes were –16.3, –13.1, –32.5, –34.3, and –38.8 kcal/mol, respectively, again surpassing the binding energies of metformin to the same targets. Correspondingly, the calculated inhibition constants (Ki) for FA were 1.09 × 10^–12^ M for α-amylase, 2.9 × 10^–10^ M for α-glucosidase, 1.42 × 10^–24^ M for fructose-1,6-bisphosphatase, 6.76 × 10^–26^ M for glucose-6-phosphatase, and 3.37 × 10^–29^ M for glycogen synthase kinase-3β — demonstrating much stronger binding affinities compared to metformin. The key interaction modes between FA and the active sites of the target proteins primarily involved hydrogen bonding. Specifically, FA formed hydrogen bonds with TRP-59, GLY-63, ARG-195, and HIS-299 in α-amylase; ASP-542 in α-glucosidase; CYS-179 in fructose-1,6-bisphosphatase; ASP-30, TYR-44, and LYS-329 in glucose-6-phosphatase; and VAL-135 and ASP-200 in glycogen synthase kinase-3β.

**Table 1:** Docking analysis of ferulic acid against carbohydrate-metabolising enzymes

Target protein	Compound	Docking score [kcal/mol]	MM-GBSA binding energy [kcal/mol]	Inhibition constant [M]
α-amylase	Ferulic acid	–6.5	–16.3	1.09 × 10^–12^
	Metformin	–2.0	–0.9	0.21
α-glucosidase	Ferulic acid	–4.1	–13.1	2.9 × 10^–10^
	Metformin	–1.7	4	861
Fructose-1,6-bisphosphatase	Ferulic acid	–5.7	–32.5	1.42 × 10^–24^
	Metformin	–3.2	–20.0	1.07 × 10^–15^
Glucose-6-phosphatase	Ferulic acid	–5.1	–34.0	6.76 × 10^–26^
	Metformin	–2.4	–11.24	1.09 × 10^–09^
Glycogen synthase kinase-3β	Ferulic acid	–7.6	–38.8	3.37 × 10^–29^
	Metformin	–2.8	–14.2	1.63 × 10^–11^

**Figure 10:**
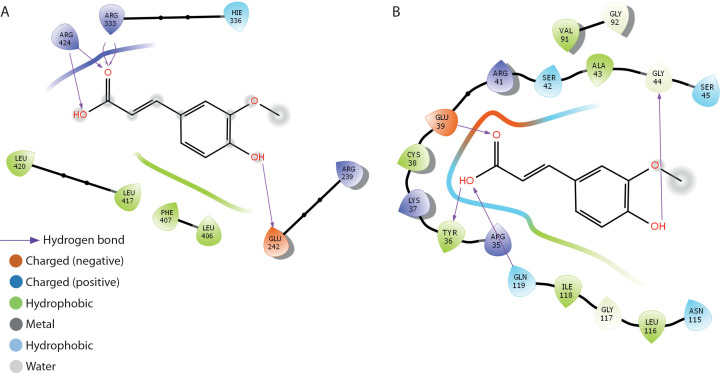
2D binding interaction between ferulic acid and oxidative stress and inflammation mediators. **A**) myeloperoxidase. **B**) NF-kB-p65

#### Oxidative stress and inflammation mediators

The binding interactions of FA with myeloperoxidase and NF-κB-p65 are presented in Table 2 and Figure 10A–B. FA exhibited strong binding affinities, with docking scores of –5.2 and –6.6 kcal/mol for myeloperoxidase and NF-κB-p65, respectively, outperforming metformin. The estimated free binding energies for FA with myeloperoxidase and NF-κB-p65 were –32.6 and –38.9 kcal/mol, respectively. The corresponding inhibition constants (Ki) were 1.19 × 10^–24^ M for myeloperoxidase and 2.24 × 10^–29^ M for NF-κB-p65, indicating much stronger binding affinities than those of metformin. The key interaction modes between FA and the amino acid residues within the active sites of the target proteins were primarily through hydrogen bonding. Specifically, FA formed hydrogen bonds with GLU-242, ARG-333, and ARG-424 in myeloperoxidase, and with TYR-36, GLN-44, and GLY-199 in NF-κB-p65.

**Table 2:** Docking analysis of ferulic acid against oxidative stress and inflammation mediators

Target protein	Compound	Docking score [kcal/mol]	MM-GBSA binding energy [kcal/mol]	Inhibition constant [M]
Myeloperoxidase	Ferulic acid	–5.2	–32.6	1.19 × 10^–24^
	Metformin	–3.1	–14.5	2.29 × 10^–11^
Nuclear factor κB p65	Ferulic acid	–6.6	–38.9	2.84 × 10^–29^
	Metformin	–2.8	–21.2	2.34 × 10^–16^

## Discussion

Our study demonstrated that the combination of fructose and STZ significantly impaired pancreatic β-cell function, leading to disrupted insulin signaling. This dysfunction subsequently resulted in hyperglycemia and elevated levels of glycosylated hemoglobin in male Wistar rats. In addition to regulating glucose homeostasis, insulin plays a crucial role in protein metabolism by promoting protein synthesis and inhibiting excessive protein degradation. Its deficiency – strongly associated with β-cell dysfunction – can lead to reduced levels of total protein, including albumin and globulin (Rasch and Mogensen 1980; Stumvoll et al. 2005). In our study, treatment of type 2 diabetic rats with varying doses of FA for 28 days significantly improved pancreatic β-cell function, as evidenced by reduced serum glucose levels, increased serum insulin concentration, decreased glycosylated hemoglobin, and elevated total serum protein levels compared to the untreated group. Weight loss is a common feature of T2D, primarily driven by increased muscle and fat breakdown, excessive urination (polyuria), and glucose loss through urine (glycosuria) (Galicia-Garcia et al. 2020).

In this study, the induction of T2D in male Wistar rats using fructose and STZ resulted in progressive weight loss. Interestingly, oral administration of FA for 28 days significantly reversed this effect in a dose-dependent manner, suggesting that FA may enhance metabolism and overall physiological health. The liver plays a central role in maintaining glucose balance and regulating overall energy metabolism. Therefore, protecting it from diabetes-induced damage is essential for effective diabetes management. Serum levels of enzymes such as AST, ALT, and ALP are key markers of liver dysfunction and are often elevated in conditions like T2D (Tanase et al. 2020). Our findings revealed that diabetic rats exhibited elevated serum levels of AST, ALT, and ALP, indicative of hyperglycemia-induced hepatic injury. In contrast, FA treatment significantly reduced these enzyme levels compared to untreated diabetic rats, highlighting its hepatoprotective effects. This observation is consistent with previous findings demonstrating that FA can protect the liver from hyperglycemia-induced damage (Salau et al. 2023).

The kidney also plays a critical role in maintaining glucose balance and overall metabolic function. However, diabetic conditions can lead to significant renal impairment, which is detectable by elevated serum concentrations of creatinine, uric acid, and BUN (Alqahtani et al. 2022), as corroborated in this study. Notably, our results show that treatment with FA significantly decreased the serum levels of these kidney biomarkers, strongly suggesting a protective effect of FA against T2D-induced renotoxicity. T2D is characterized by altered carbohydrate metabolism, primarily due to defective insulin production and function (Jiang et al. 2020). In addition to improving insulin production, targeting key enzymes involved in carbohydrate metabolism represents a promising strategy for diabetes management. The enzymes α-amylase, primarily found in the pancreas, and α-glucosidase, mainly located in the small intestine, are responsible for breaking down dietary carbohydrates into glucose. α-Amylase cleaves α-D-(1,4)-glycosidic bonds in carbohydrates to produce oligosaccharides, which are subsequently broken down into simple glucose units by α-glucosidase (Jiang et al. 2020).

Inhibiting these enzymes offers an effective strategy for diabetes management by slowing the postprandial rise in blood glucose levels following carbohydrate-rich meals. Our study found that FA-treated diabetic rats exhibited reduced pancreatic α-amylase and intestinal α-glucosidase activities compared to untreated diabetic rats, thereby highlighting the antidiabetic potential of FA. Hexokinases catalyze the conversion of glucose to glucose-6-phosphate, the first step in glycolysis. Reduced expression and activity of hexokinases have been implicated in the onset of diabetes (Braithwaite et al. 1995). In this study, FA-treated diabetic rats exhibited higher hepatic hexokinase activity than untreated diabetic rats, suggesting FA’s potential to promote hexokinase activity and support glucose metabolism. In individuals with T2D, gluconeogenesis is a major source of endogenous glucose production (Hatting et al. 2018). Therefore, inhibiting key mediators of gluconeogenesis – such as glucose-6-phosphatase and fructose-1,6-bisphosphatase – alongside enhancing insulin production (which naturally suppresses gluconeogenic enzymes), may represent an effective approach to diabetes management.

By targeting these enzymes, excessive glucose production contributing to hyperglycemia in T2D can be reduced, thereby improving overall glycemic control. Notably, our study showed that diabetic rats treated with FA exhibited reduced hepatic glucose-6-phosphatase and fructose-1,6-bisphosphatase activities compared to the untreated diabetic group, highlighting FA’s potential to modulate gluconeogenesis and support glycemic control. Glycogen is the primary storage form of glucose in cells. In T2D, glycogen content is often reduced due to impaired glucose uptake and synthesis in the liver and skeletal muscles (Galicia-Garcia et al. 2020). In our study, FA treatment led to increased hepatic glycogen levels in diabetic rats compared to untreated controls. This effect may be attributed to the inhibition of glycogen synthase kinase-3β (GSK-3β), a negative regulator of glycogen synthesis. Inhibiting this enzyme can enhance glycogen storage in the liver and muscles, improving blood glucose control and insulin sensitivity (Teli and Gajjar 2023). Oxidative stress arises when reactive oxygen species (ROS) and reactive nitrogen species (RNS) overwhelm the body’s antioxidant defense system, leading to free radical–mediated cellular damage and impaired function (Sies et al. 2022). This phenomenon is implicated in the pathogenesis of several chronic diseases, including T2D (Ceriello 2003). In our study, administration of fructose and STZ induced T2D in male Wistar rats, which was accompanied by oxidative stress in the liver and kidney. This was evidenced by a marked reduction in superoxide dismutase (SOD) and catalase (CAT) activities, decreased glutathione (GSH) levels, and elevated malondialdehyde (MDA) concentrations in these tissues.

Interestingly, treatment with varying doses of FA significantly attenuated hepatic and renal oxidative stress in a manner comparable to metformin, thereby underscoring the antioxidant potential of FA. Plant polyphenols such as FA are well known for their potent antioxidant effects, primarily through the neutralization of ROS and RNS – major contributors to oxidative stress (Hano and Tungmunnithum 2020). Histopathological analysis further supported the protective effects of FA on the liver and kidney against oxidative stress – and inflammationinduced damage in T2D. Untreated diabetic rats exhibited liver congestion, central vein dilation, and nuclear fragmentation, while kidney tissues showed glomerular degeneration, enlargement of the urinary space, and degeneration of convoluted tubule cells — features that compromise organ function. However, treatment with FA for 28 days markedly reversed these histological alterations, highlighting its reparative potential.

Oxidative stress and inflammation are closely intertwined in diabetes, where elevated levels of ROS not only activate inflammatory pathways but also exacerbate insulin resistance and pancreatic β-cell dysfunction (Keane et al. 2015). This creates a self-perpetuating cycle that accelerates the progression of diabetic complications. Hyperglycemia further aggravates this process by significantly upregulating pro-inflammatory cytokines such as NF-κB-p65, IL-1β, and IL-6, thus reinforcing chronic inflammation and contributing to diabetes-related pathologies (Vasbinder et al. 2022). This reciprocal relationship between hyperglycemia and inflammation underscores the need for therapeutic strategies that target both biochemical processes as part of comprehensive diabetes management. In this context, our study demonstrated that treatment of type 2 diabetic rats with FA for 28 days significantly downregulated hepatic and renal levels of key pro-inflammatory mediators – including NF-κB-p65, IL-1β, and IL-6 – compared to untreated diabetic rats. This finding aligns with previous studies (Adeyi et al. 2023; Park and Han 2024), highlighting the potent anti-inflammatory properties of FA and suggesting its potential as a promising therapeutic agent for mitigating the inflammatory burden of diabetes and its associated complications.

Interestingly, our findings align with previous studies demonstrating that FA can ameliorate diabetic conditions by enhancing insulin signaling, improving kidney and liver function, reducing dyslipidemia, and modulating key diabetogenic enzymes in diabetic rat models (Roy et al. 2013; Salau et al. 2023). While these earlier studies primarily focused on its effects in pancreatic tissue, our study is the first to report that FA at a dose of 50 mg/kg bw confers protective effects on the liver and kidney in T2D. This novel finding expands the current understanding of FA’s therapeutic potential beyond pancreatic protection by demonstrating its capacity to safeguard vital organs commonly affected by diabetic complications. Specifically, our results highlight the ability of FA to simultaneously mitigate oxidative stress, lipid peroxidation, and inflammatory responses in the liver and kidney of a fructose–STZ-induced type 2 diabetic rat model. By targeting these pathological mechanisms, FA may play a critical role in preventing diabetes-associated hepatic and renal dysfunction, thereby underscoring its potential as a multifaceted therapeutic agent for the management of T2D and its complications.

To further confirm the antidiabetic mechanisms of FA, molecular docking analysis was conducted. The results showed that FA exhibited stronger inhibitory potential against α-amylase and α-glucosidase compared to metformin. Inhibitors of α-amylase and α-glucosidase exert their antidiabetic effects by reversibly occupying the substrate-binding sites of these enzymes, thereby reducing the binding and breakdown of starch and other dietary carbohydrates. This action slows the digestion and absorption of carbohydrates in the gastrointestinal tract and intestines (Gong et al. 2020). Additionally, FA demonstrated a stronger inhibitory effect on fructose-1,6-bisphosphatase and glucose-6-phosphatase than metformin. This suggests that FA may effectively suppress gluconeogenesis, preventing glucose production from noncarbohydrate sources such as amino acids and lactate, thereby contributing to glucose homeostasis. Additionally, FA exhibited a stronger inhibitory effect on GSK-3β than metformin, suggesting that it can bind to and inhibit GSK-3β, thereby preventing the phosphorylation of glycogen synthase. This inhibition would lift the repressive effect of GSK-3β on glycogen synthase, promoting glycogen synthesis – a beneficial outcome for diabetes treatment. FA also exhibited stronger inhibitory potential against myeloperoxidase compared to metformin. Myeloperoxidase, an enzyme involved in the generation of hypochlorous acid (HOCl), significantly contributes to the production of ROS (Ndrepepa 2019). Thus, the binding of FA to myeloperoxidase may inhibit its activity, potentially protecting cells from oxidative stress-related damage and further supporting the role of FA in mitigating oxidative stress in diabetic conditions. Moreover, FA showed stronger inhibitory potential against NF-κB-p65 compared to metformin. Inhibiting NF-κB-p65 could prevent its translocation from the cytoplasm to the nucleus, where it functions as a transcription factor for pro-inflammatory mediators such as IL-1β and IL-6 (Serasanambati and Chilakapati 2016).

## Conclusions

This study underscores the broad antidiabetic potential of FA. It improved β-cell function and glucose metabolism, and offered liver and kidney protection through antioxidative and anti-inflammatory actions. Molecular docking further supported the strong inhibitory potential of FA on key enzymes involved in carbohydrate metabolism, oxidative stress, and inflammation — surpassing the effects observed with metformin. Collectively, these findings highlight FA as a promising candidate for the management of T2D and its associated complications.

## Supplementary Material

Hepato-renal protection by ferulic acid in a type 2 diabetic rat model: *in vivo* and *in silico* insights into carbohydrate metabolism, REDOX balance, and inflammation modulation
