# Supplementary material for: Hepato-renal protection by ferulic acid in a type 2 diabetic rat model: in vivo and in silico insights into carbohydrate metabolism, REDOX balance, and inflammation modulation
Source: BioTechnologia (Pozn). 2025 Sep 7;106(3):309–26. doi: 10.5114/bta/207911 (PMC12550677; doi:10.5114/bta/207911)
Supplement: Hepato-renal protection by ferulic acid in a type 2 diabetic rat model: in vivo and in silico insights into carbohydrate metabolism, REDOX balance, and inflammation modulation [file BTA-106-3-207911-s001.pdf]

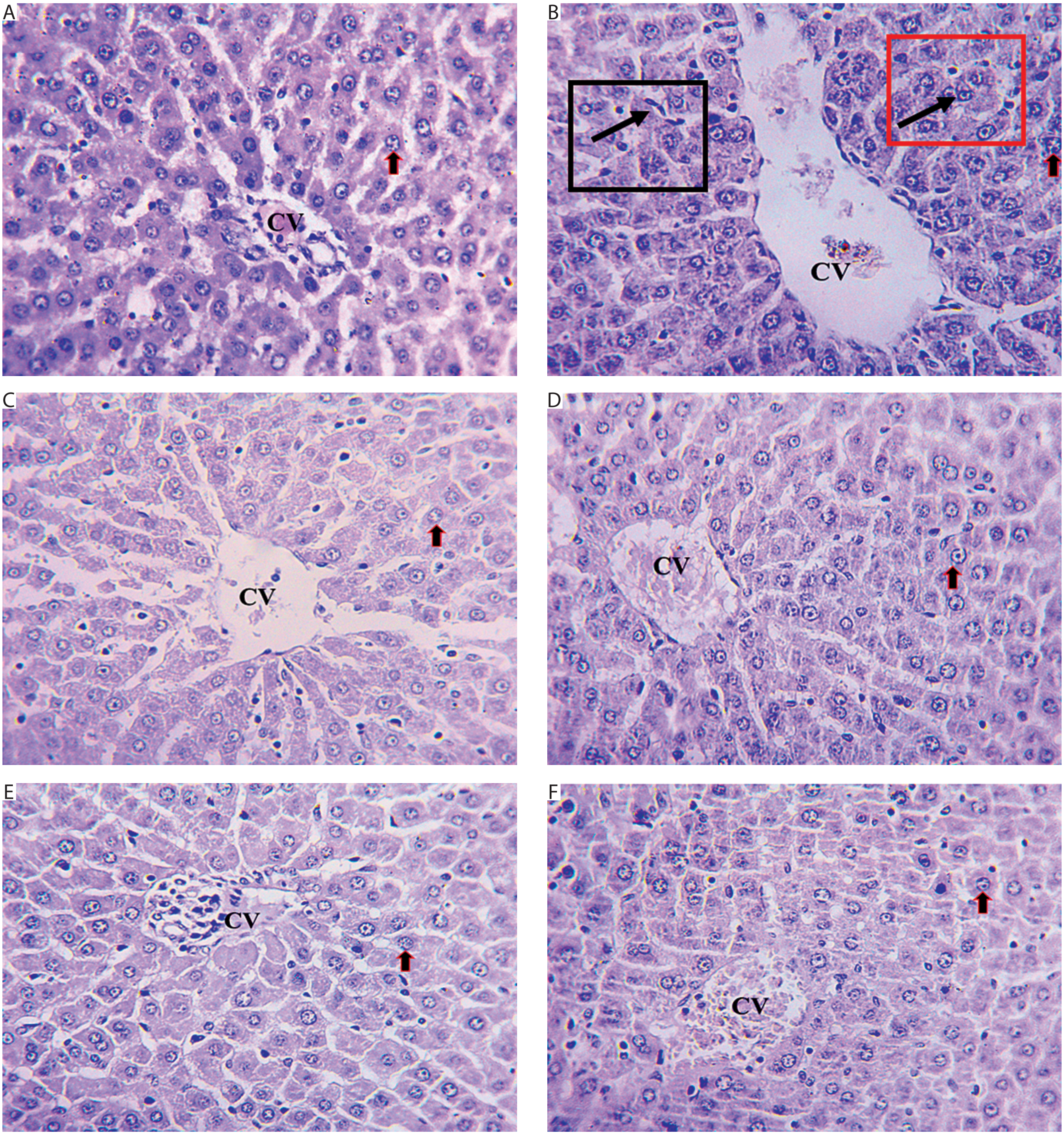

Supplementary Figure 1. Representative photomicrograph images of the liver of rats. **A**) Normal control group showing no blood congestion, moderate vacuolation, and no visible lesions. **B**) Untreated diabetic group showing congestion, dilation of the central vein, vacuolar degeneration (black arrow in black box), and degenerated binucleated hepatocytes (black arrow in red box). **C**) Diabetic group treated with 25 mg/kg bw ferulic acid showing mild congestion with no nuclear fragmentation. **D**) Diabetic group treated with 50 mg/kg bw ferulic acid showing no blood congestion, mild vacuolation, and moderate loss of hepatocytes. **E**) Normal rats administered only 50 mg/kg bw ferulic acid showing moderate congestion and moderate loss of hepatocytes. **F**) Diabetic group treated with 200 mg/kg bw metformin showing moderate congestion and mild degeneration of hepatocytes. All stained with H&E (mag. ×800). Black arrow – hepatocytes, CV – central vein

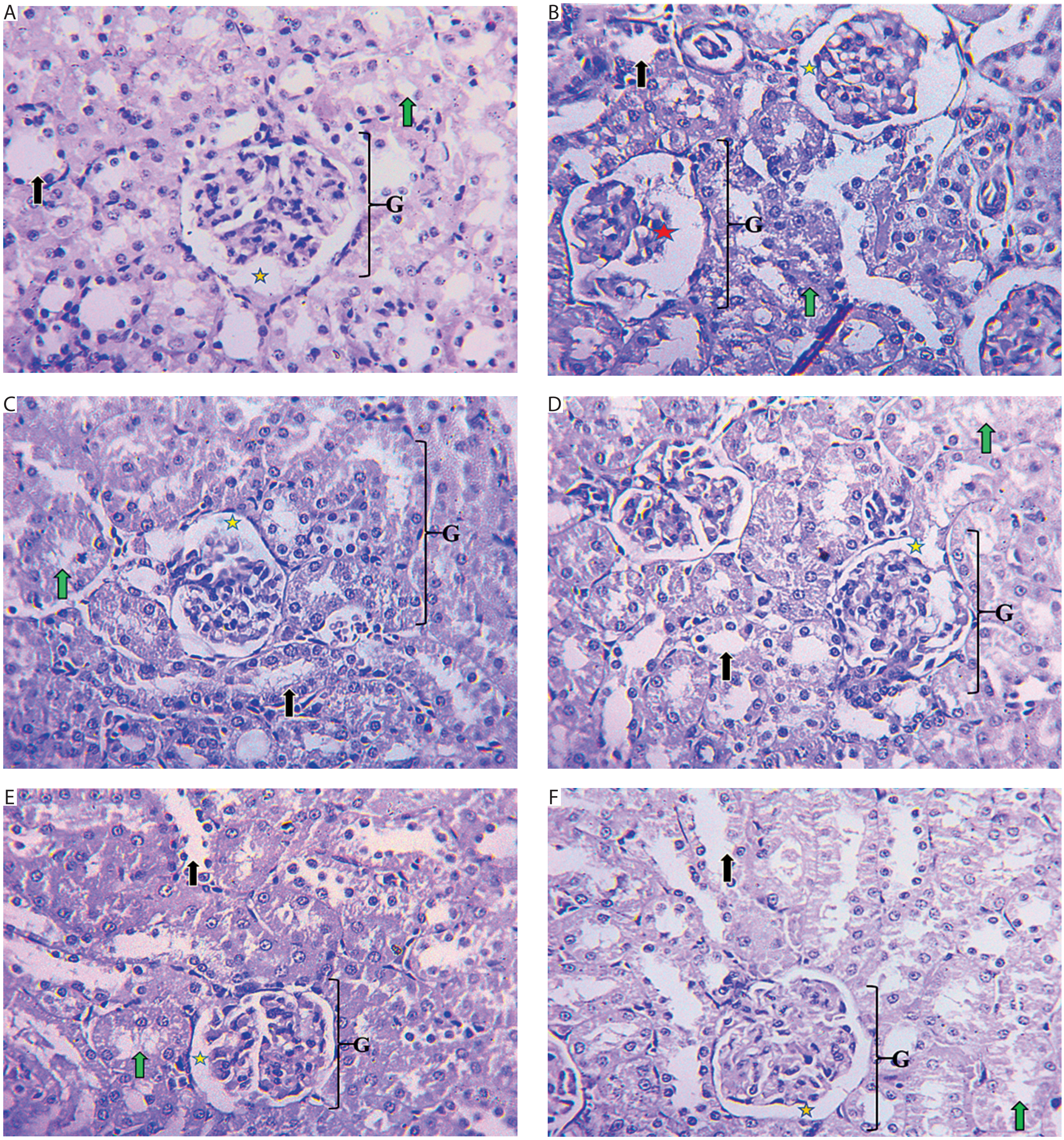

Supplementary Figure 2. Representative photomicrograph images of the kidney of rats. **A**) Normal control group showing normal architecture of the glomerulus and convoluted tubules with no visible lesions. **B**) Untreated diabetic group showing severe degeneration of the glomerulus, enlargement of the urinary space, and degeneration of convoluted tubule cells. **C**) Diabetic group treated with 25 mg/kg bw ferulic acid showing shrunken glomerulus and moderate loss of convoluted tubules. **D**) Diabetic group treated with 50 mg/kg bw ferulic acid showing moderate degeneration of the glomerulus and convoluted tubules. **E**) Normal group administered 50 mg/kg bw ferulic acid showing moderate loss of convoluted tubules. **F**) Diabetic group treated with 200 mg/kg bw metformin showing moderate degeneration of the glomerulus and convoluted tubules. All stained with H&E (mag. ×800). G – glomerulus, black arrow – distal convoluted tubules, green arrow – proximal convoluted tubules, yellow star – urinary space
